# Climate Change Dependence in Ex Situ Conservation of Wild Medicinal Plants in Crete, Greece

**DOI:** 10.3390/biology12101327

**Published:** 2023-10-11

**Authors:** Michael Bariotakis, Luciana Georgescu, Danae Laina, Margianna Koufaki, Maria Souma, Sotirios Douklias, Konstantinos A. Giannakakis, Kyriaki N. Chouli, Luca Paoli, Stefano Loppi, Reggina Karousou, Petr Smykal, Elias Castanas, Stergios A. Pirintsos

**Affiliations:** 1 Department of Biology, University of Crete, 714 09 Heraklion, Greece; navaak@gmail.com (M.B.); luciana_georgescu@yahoo.com (L.G.); danae.31@hotmail.com (D.L.); koufakimargianna@gmail.com (M.K.); maria.souma@outlook.com (M.S.); doukliassotiris@gmail.com (S.D.); bio3523@edu.biology.uoc.gr (K.A.G.); bio3512@edu.biology.uoc.gr (K.N.C.); 2 Department of Biology, University of Pisa, 56126 Pisa, Italy; luca.paoli@unipi.it; 3 Department of Life Sciences, University of Siena, 53100 Siena, Italy; stefano.loppi@unisi.it; 4 School of Biology, Aristotle University of Thessaloniki, 541 24 Thessaloniki, Greece; karousou@bio.auth.gr; 5 Department of Botany, Palacký University Olomouc, 783 71 Olomouc, Czech Republic; petr.smykal@upol.cz; 6 School of Medicine, University of Crete, 714 09 Heraklion, Greece; castanas@uoc.gr; 7 Botanical Garden, University of Crete, Gallos University Campus, 741 00 Rethymnon, Greece

**Keywords:** medicinal plants, wild harvest, precision agriculture, ex situ conservation, climate change, Ecological Niche Modeling, Crete, Lamiaceae, habitat shift, adaptation strategies

## Abstract

**Simple Summary:**

Over 80% of people globally rely on traditional medicine, primarily using medicinal plants, leading to rising demand both domestically and internationally. This has impacted natural ecosystems due to wild harvesting. Cultivating these medicinal species has been proposed as a conservation solution. However, in today’s climate change-focused world, smallholder farmers face uncertainty regarding how cultivation benefits match up against climate impacts, increasing their stress. The effects of climate change on the ex situ cultivation of ten significant medicinal plants in Crete, Greece, were analyzed by predicting their habitat suitability in future climates. The findings showed varied effects across species, categorizing them into three based on potential cultivation area changes. This data, showing where these areas might increase or decrease, can guide regional management strategies to assist practitioners.

**Abstract:**

Over 80% of the global population addresses their primary healthcare needs using traditional medicine based on medicinal plants. Consequently, there’s a rising demand for these plants for both household and industrial use at local, regional, national, and international levels. However, wild harvesting has negatively impacted natural ecosystems. Cultivating medicinal species has been proposed as a conservation strategy to alleviate this pressure. Yet, in this age of global climate change concerns, smallholder farmers’ views on the benefits of such cultivation clash with the uncertainties of climate change impacts, amplifying their anxieties. In this context, the climate change dependence of ex situ cultivation of ten wild medicinal taxa with significant ethnopharmacological interest in Crete, Greece, were studied, projecting their potential habitat suitability under various future climate scenarios. The results demonstrated species-specific effects. Based on the potential cultivation area gains and losses, these effects can be categorized into three groups. We also outlined the spatial patterns of these gains and losses, offering valuable insights for regional management strategies benefiting individual practitioners.

## 1. Introduction

The World Health Organization (WHO) estimates that traditional medicine, primarily rooted in medicinal plants, meets the primary healthcare needs of over 80% of the global population [1].

Furthermore, a variety of products derived from medicinal plants range from raw materials to refined and packaged goods. These include medicines, herbal remedies, teas, beverages (both alcoholic and non-alcoholic), cosmetics, confections, dietary supplements, varnishes, and insecticides [2]. Consequently, there is a persistent and escalating demand for medicinal plants for both household and industrial applications on local, regional, national, and international scales [2,3,4,5,6]. This demand is projected to grow by 10–20% annually, with over 70% of these plants being sourced from the wild [7].

Natural ecosystems have undeniably suffered due to such wild harvesting [8]. Propagating and cultivating medicinal species in controlled settings has been advocated for some time as a strategy to alleviate this environmental pressure [1,9].

Farmers’ perceptions of the benefits offered by various precision agriculture technologies vary widely [10]. Despite the challenges posed by traditional agriculture, many farmers are reluctant to transition to these new methods. Two primary reasons underlie this hesitation: (a) their comfort and familiarity with traditional farming practices and (b) the long-standing proven reliability of traditional agriculture in terms of its consistent performance over centuries.

Transitioning from traditional to precision agriculture introduces a sense of uncertainty, especially among smallholder farmers. This unease stems from their reliance on specialized scientific knowledge, leading to a dependence on external experts and resources, which they feel are beyond their control.

To address this barrier, numerous initiatives have been launched [11,12], providing accessible expertise on precision agriculture, particularly via online platforms. These efforts aim to assist smallholder farmers and familiarize them with the specialized knowledge required.

An example of such an effort concerns previous research on cultivating wild medicinal plants in Crete. A number of taxa with known ethnopharmacological uses [13] were studied, which have been employed extensively in traditional medicine and as culinary supplements. The data propose potential habitats suitable for these taxa across the study area in the context of precision agriculture. In addition, a platform has been developed where all interested groups can access and utilize this specialized knowledge, from the policy-maker to the individual practitioner level (https://navaak.shinyapps.io/agriSuitNew/ accessed on 30 August 2023

Regarding the second reason, it is worth mentioning that, in the era of global concern about climate change [14], the unknown response of the medicinal plant cultivations to climatic scenarios strongly increases the anxiety of the smallholder farmers, especially due to the new activity’s future duration, which has to be known to develop their business plan. In the Mediterranean basin, this anxiety is reinforced and may also derive from the inter-annual variability in seasonal weather, a characteristic of the Mediterranean climate, which every year generates uncertainty in the decision-making processes of cultivation practice in their field [15].

The present work attempts (a) to study the climate change dependence of ex situ cultivation of ten wild medicinal taxa of high ethnopharmacological interest across the islands of Crete, Greece, projecting the potential habitat suitability in different future climate scenarios, and (b) to explore the expected spatial patterns for possible management implications.

## 2. Materials and Methods

### 2.1. Medicinal Plants and Study Area

Ten wild plant species known for their medicinal properties [16,17,18,19,20,21,22,23], all native to Crete, were examined. These include *Calamintha nepeta* (L.) Savi subsp. *glandulosa* (Req.) P.W. Ball, *Thymbra capitata* (L.) Cav., *Melissa officinalis* L., *Micromeria juliana* (L.) Rchb., *Origanum dictamnus* L., *Origanum vulgare* L. subsp. *hirtum* (Link) Ietsw., *Origanum onites* L., *Salvia fruticosa* Mill., *Salvia pomifera* L. subsp. *pomifera*, and *Satureja thymbra* L., with *Origanum dictamnus* L. existence endemic to the island. The nomenclature of the Greek taxa studied is after Flora of Greece Web [24].

Crete is the largest island of Greece, with a total surface of 8729 km^2^ and a west–east extension of about 254 km, where four mountain ranges (Lefka Ori, Psiloritis—including Kedhros, Dikti, and Afendis Kavousi) occur [25]. The island shares the same latitude with central Tunisia. The elevation and longitude have the most significant influence on precipitation and yield the highest spatial correlation (positive to elevation and negative to longitude) [26]. For example, for the period 1974–2005, areal mean annual precipitation is estimated to 750 mm and varies from ca. 440 mm in the east (Ierapetra; 10 m a.s.l) to ca. 2120 mm in the west (Askifou; 740 m a.s.l). Notably, the eastern part of the island of Crete has been characterized as a major hot spot in the Mediterranean zone concerning drought susceptibility [27,28].

### 2.2. Data Collection

A comprehensive survey was carried out at 665 locations on the island, noting the occurrence of the mentioned plant species. The chosen sites were randomly selected, considering the island’s geography. The data collection were thorough, stemming from extensive field visits that encapsulated the great majority of each plant species’ known habitat. Previous knowledge, such as [29], ensured that no significant habitats were overlooked. Since these species are easily identifiable and non-cryptic, there’s a minimal chance of missing them. The study revealed a range of occurrences for each species, with counts ranging from 24 (for *Salvia pomifera* subsp. *pomifera*) to 301 (for *Thymbra capitata*), reflecting their respective abundances on the island.

The bioclimatic variables with a spatial resolution of 30 s (approximately 1 km^2^) were downloaded from the WorldClim database (https://worldclim.org/ accessed on 30 August 2023) [30].

### 2.3. Ecological Niche Modeling (ENM)

For over two decades, ENM has been a reliable tool in addressing diverse eco-geographical challenges, ranging from studying hybrid zones [31,32] and conserving Tertiary relict species [25,33] to understanding the macroecology of Crop Wild Relatives (CWR) [34] and from the management of invasions in the face of climate change [35] to the response of cultivated species to climate change [36].

For predicting the potential niche of the species in focus [13], MaxEnt version 3.4.1 [37,38], a version implemented in R using the *dismo* package, was utilized. This machine-learning method hinges on the principle of maximum entropy. MaxEnt stands out for its strong predictive capabilities, even with limited data entries [39,40]. Moreover, as a presence-only modeling technique, it estimates a taxon’s potential niche (which aligns perfectly with this study’s objectives) instead of its realized niche [41].

As a measure of model performance, the area under the ROC curve (AUC) was calculated. This was performed by retaining 10% of the presence points as the test sample and iterating this process 10 times per model. After the process was completed, the mean AUC of those 10 iterations was calculated. As this process was performed purely for validation purposes, the final models presented and discussed in this work were trained with 100% of the available presence records.

### 2.4. Future Climate Scenarios

Future climate scenarios were calculated using the global climate model MIROC-6 (Model for Interdisciplinary Research on Climate-6), developed by the Japan Agency for Marine-Earth Science and Technology (JAMSTEC), Atmosphere and Ocean Research Institute (AORI), University of Tokyo, and National Institute for Environmental Studies (NIES), Japan [42,43]. This is a newly developed climate model, with updates to its physical parameterizations in all sub-modules, and it was utilized to predict the future potential distribution of the species for the years 2040, 2060, and 2080 with the two Shared Socio-Economic Pathways (SSPs) (SSP 126 and SSP 370).

SSP-based scenarios further refine the previous greenhouse gas concentration scenarios known as Representative Concentration Pathways or RCPs. The SSPs are based on five narratives (SSP 1-SSP 5) describing alternative socio-economic developments, including sustainable development, regional rivalry, inequality, fossil-fueled development, and middle-of-the-road development. SSP 1 envisions relatively optimistic trends for human development, while SSP 3 is more pessimistic. Specifically, SSP 126 with 2.6 W/m² by the year 2100 is a remake of the optimistic scenario RCP2.6 and was designed with the aim of simulating a development that is compatible with the 2 °C target.

This scenario also presumes the implementation of climate protection measures. The SSP 370, projecting 7 W/m² by the year 2100, falls within the upper-middle spectrum of all scenarios.

It was newly introduced after the RCP scenarios, closing the gap between RCP6.0 and RCP8.5 (for an overview, see [44]).

In addition, future climate scenarios using Australia’s national climate model ACCESS-2 (Australian Community Climate and Earth Systems Simulator) were also calculated. ACCESS was initially created to focus on the Southern Hemisphere but later developed into a world-class climate model used internationally [45,46,47]. This model was used only for the year 2060 as complementary to MIROC-6 to obtain an indication of the uncertainty and limits of climatic projections in the future.

Moreover, to help estimate the propensity for change and stability of each species, all suitability maps were converted into presence/absence maps by use of the Least Training Presence threshold for each species. Then, all pixels of the study area were grouped into four categories based on the current and future predicted presence of the species: turned to suitable, remained suitable, remained unsuitable, and turned to unsuitable. Furthermore, the Stability Index was calculated as the proportion of unchanged pixels (remained suitable and remained unsuitable).

### 2.5. Data Processing and Visualization

All analysis presented in this study was performed in R version 3.6.3 [48]. Apart from the base functionality of R, some additional packages were employed, especially for the geospatial data preparation, processing, and visualization. These were *rgdal* [49], *sp* [50], and *raster* [51].

## 3. Results

Predictive performance, as indicated using mean AUC, was good (>0.8) for most of the models. As expected, a small number of models demonstrated slightly lower predictive performance (Figure S1), mainly due to the small number of records, but as Pearce et al. [52] stated, a model with an AUC value of 0.75 is considered reliably accurate.

Of course, the predictive distribution of a taxon does not accurately predict the realized distribution since taxa can be absent from suitable locations for various reasons [32,53]. Nevertheless, it is valuable as it can be used as the basis for suitability visualization and, specifically, the geographic distribution of suitability of the areas within a web-based, easy-to-use application [13].

**Figure 1:**
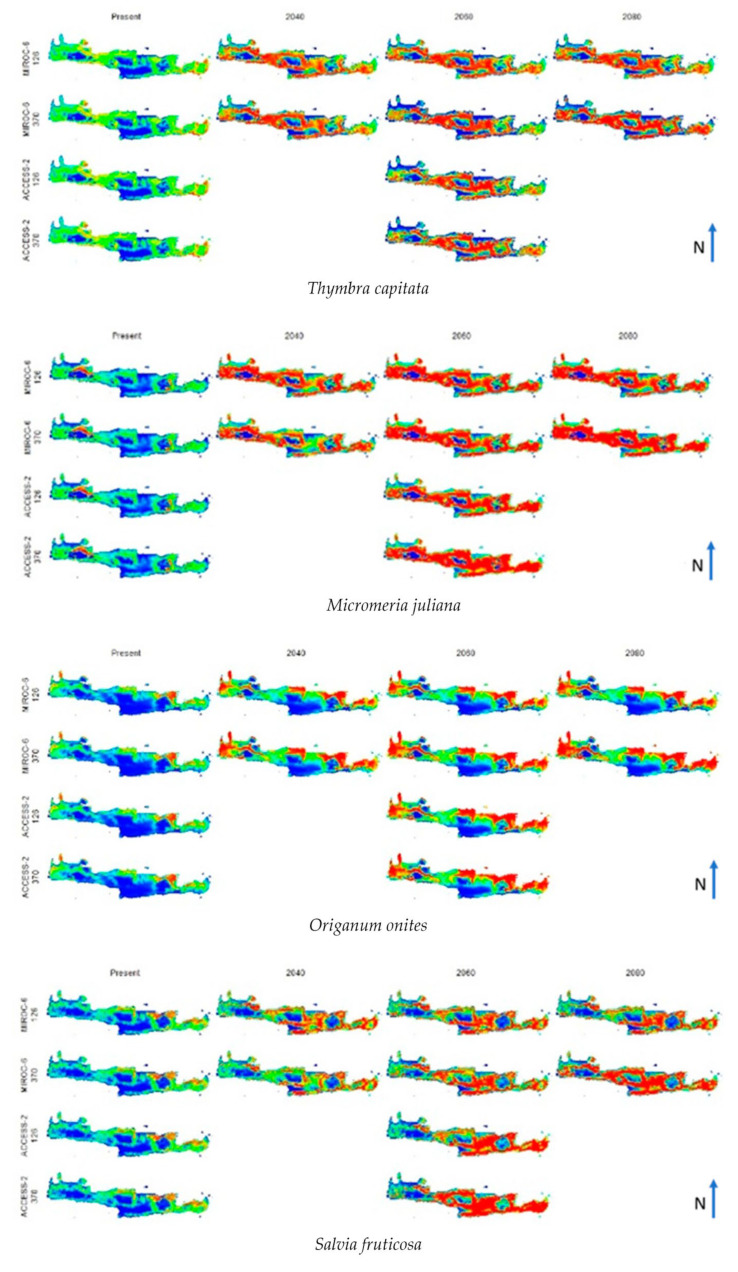

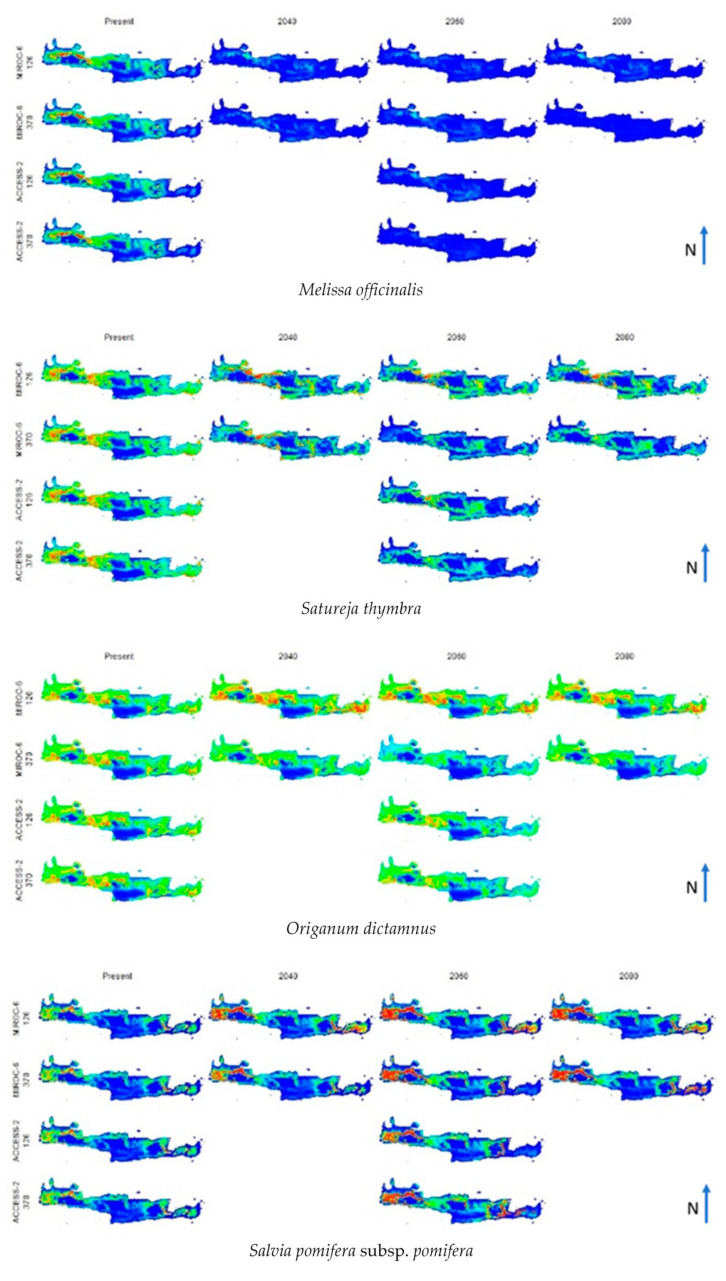

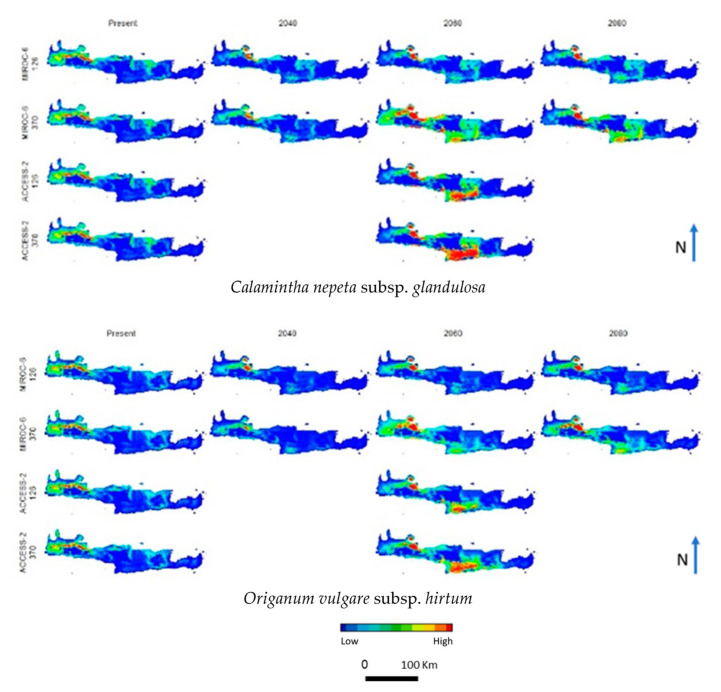
Results of MaxEnt modeling for the potential distribution of the ten studied taxa. Warmer colors represent a higher probability of occurrence scores, while colder colors represent lower probability scores. The left column corresponds to the current probabilities of occurrence, while the other columns correspond to future projections according to the year and the selected model.

Regarding future projections, in Figure 1, the outcomes resulting from the interaction of two distinct Shared Socio-economic Pathways, namely Pathway 126 and Pathway 370, coupled with two disparate climatic models, ACCESS-Australia and MIROC-Germany, for the years 2040, 2060, and 2080 are presented. It is clearly shown that the output of the ACCESS-Australia climate model is comparable with the output of MIROC-Germany both for the current and 2060 future projections and for both Shared Socio-Economic Pathways.

As shown in Figure 1, four species groups are recorded based on the projections of the future probability of occurrence. The favored group encloses four species: *Thymbra capitata*, *Micromeria juliana*, *Origanum onites*, and *Salvia fruticosa*. In this group, the probability of occurrence spatially projected to the future climate scenarios appears ameliorated compared to the current occurrence scores. The disadvantaged group includes *Origanum dictamnus*, *Melissa officinalis*, and *Satureja thymbra*. In this group, a deterioration is predicted regarding the spatial pattern of the projected probability of occurrence. Finally, the intermediate group, represented by *Calamintha nepeta* subsp. *grandulosa, Origanum vulgare* subsp. *hirtum*, and *Salvia pomifera* subsp. *pomifera*, exhibits intense spatial discontinuities between gains and losses concerning the studied future climate scenarios.

**Figure 2:**
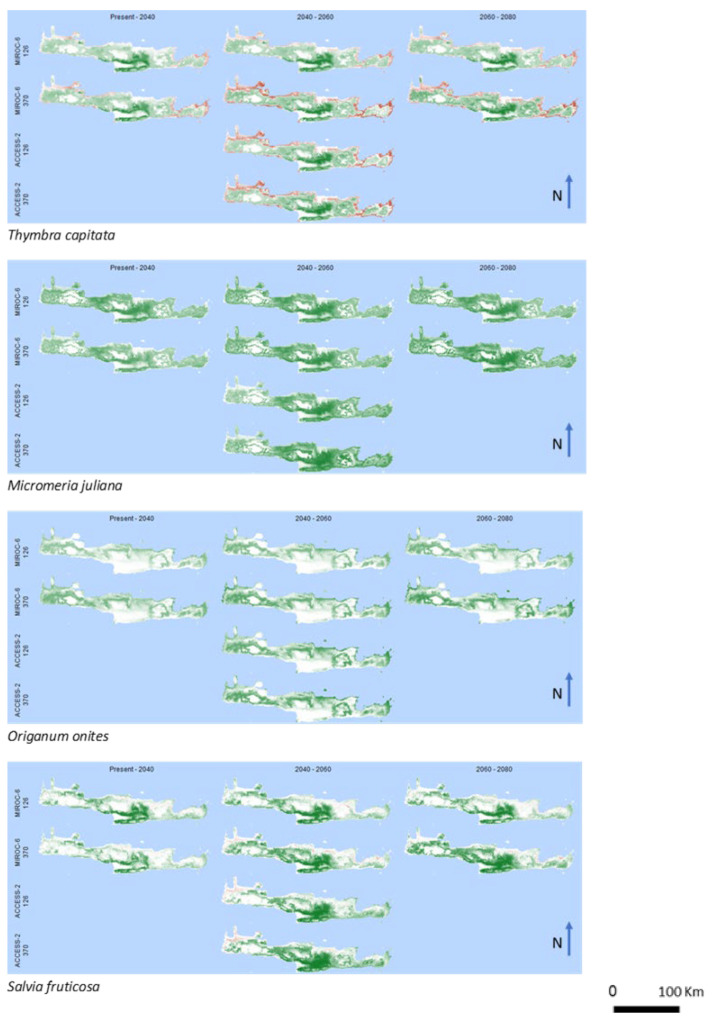
Spatial pattern of gains and losses of potential cultivated areas of the favored group for the studied years and the future climate change scenarios. Green and red colors represent gains and losses, respectively. White colors correspond to areas where no alterations are predicted.

In the case of *Thymbra capitata* (Figure 2), a member of the favored group, a significantly wide area in continental Crete, particularly in the central parts of the island and to the corresponding southern coast, represents gains of potential cultivated lands. However, losses are predicted in coastal areas, which are projected to spatially expand in the subsequent years.

On the contrary, no losses have resulted for *Micromeria juliana* (Figure 2). The projections in all studied climate scenarios predict gains of potential cultivated lands almost all over the island, excluding the high mountain areas. Similarly, no losses were predicted in the case of *Origanum onites* (Figure 2). However, the gains are not distributed all over the island and are mostly predicted on the northern coast and the western areas. No gains were predicted for the southern coast and the central-south part of the island.

In the case of *Salvia fruticosa* (Figure 2), significant gains are predicted primarily on the central-south part of the island, while no losses are expected. On the other side, in the island’s western and eastern parts, remarkably minor gains are expected.

**Figure 3:**
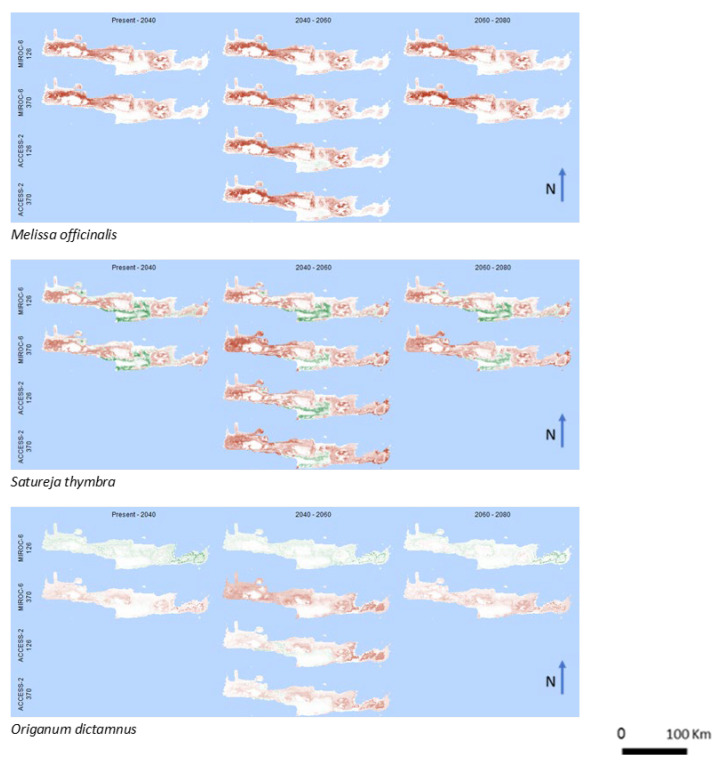
Spatial pattern of gains and losses of potential cultivated areas of the disadvantaged group for the studied years and the future climate change scenarios. Green and red colors represent gains and losses, respectively. White colors correspond to areas where no alterations are predicted.

Concerning the disadvantage group, for *Melissa officinalis* (Figure 3), all possible scenarios indicate a decrease in the projected probability of occurrence, with more pronounced losses in the northwestern part of the island, while no gains were predicted. Furthermore, a decrease in the projected probability of occurrence is expected for *Satureja thymbra* (Figure 3), with predominant losses almost all around the island. Minor gains are predicted only in the central part.

*Origanum dictamnus* (Figure 3) is the only of the studied species that reveals inconsistency between the results of the different climate scenarios. In MICRO-6 (126), only minor gains are predicted all over the island without losses, in contrast to the other three, where losses dominate with no gains, like the patterns of the other species of the same group.

**Figure 4:**
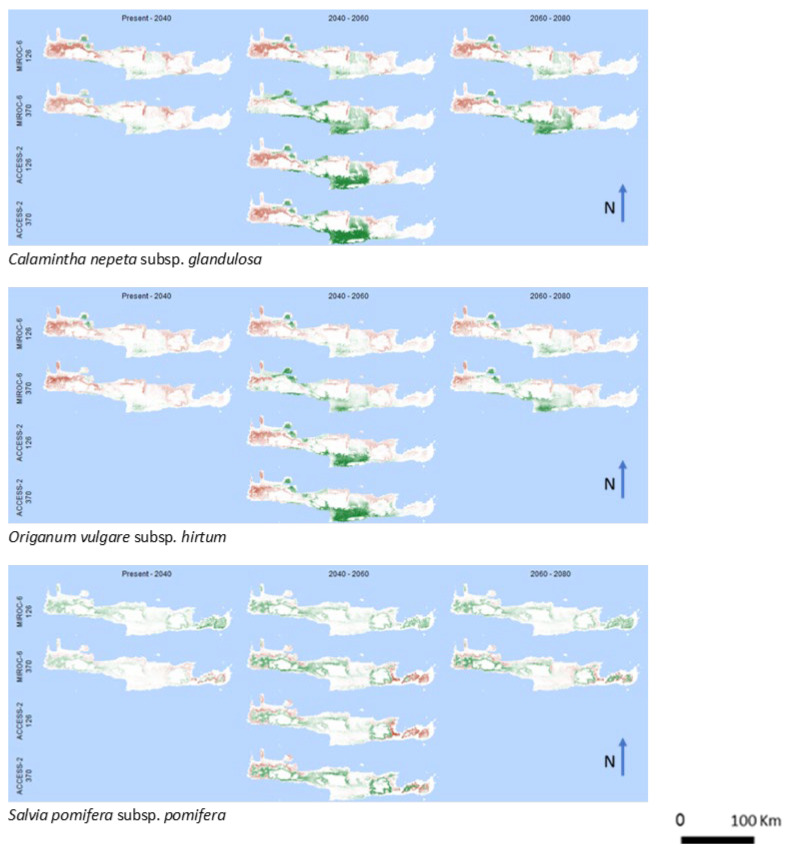
Spatial pattern of gains and losses of potential cultivated areas of the intermediate group for the studied years and the future climate change scenarios. Green and red colors represent gains and losses, respectively. White colors correspond to areas where no alterations are predicted.

*Calamintha nepeta* subsp. *grandulosa, Origanum vulgare* subsp. *hirtum,* and *Salvia pomifera* subsp. *pomifera* (Figure 4) reveals a pattern with intense spatial discontinuities between gains and losses, which are predicted to take place in the case of *Calamintha nepeta* subsp. *grandulosa* and *Origanum vulgare* subsp. *hirtum* between western and central parts, while in the case of *Salvia pomifera* subsp. *pomifera* between the eastern and western parts of the island.

**Figure 5:**
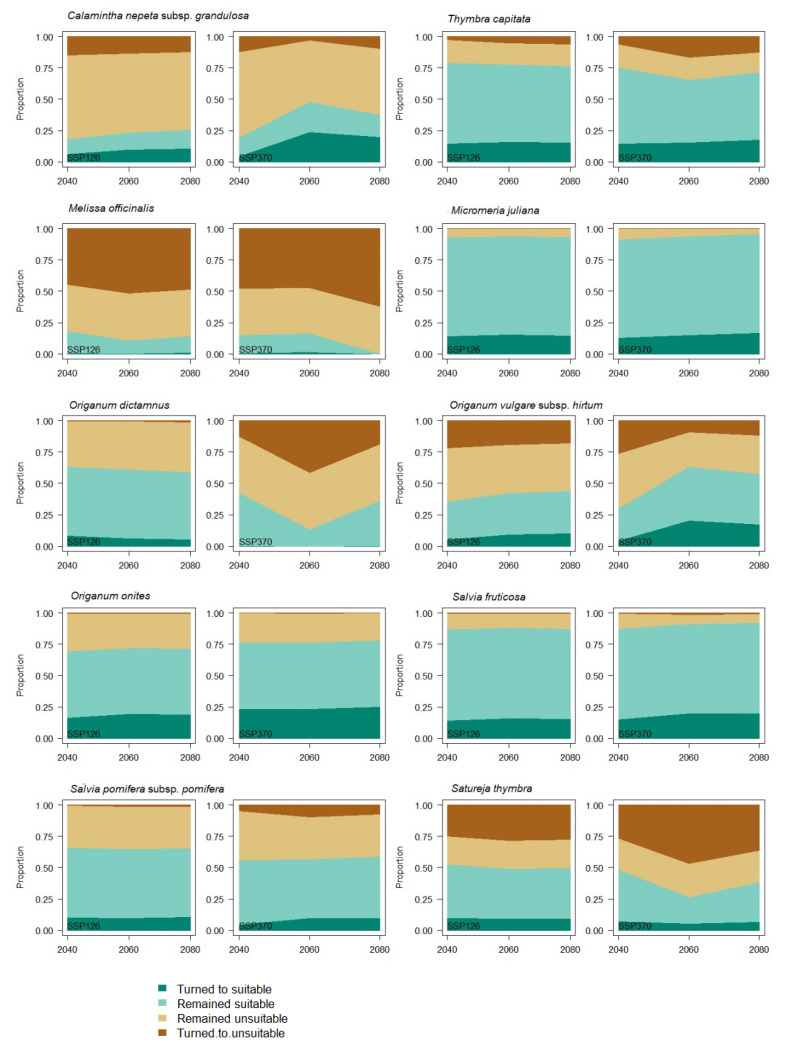
Stacked area plots of calculated cell change statistics for different years and climatic scenarios. Each colored stack represents the proportion of cells that fall into one of four categories, from bottom to top: turned to suitable, remained suitable, remained unsuitable, and turned to unsuitable.

**Figure 6:**
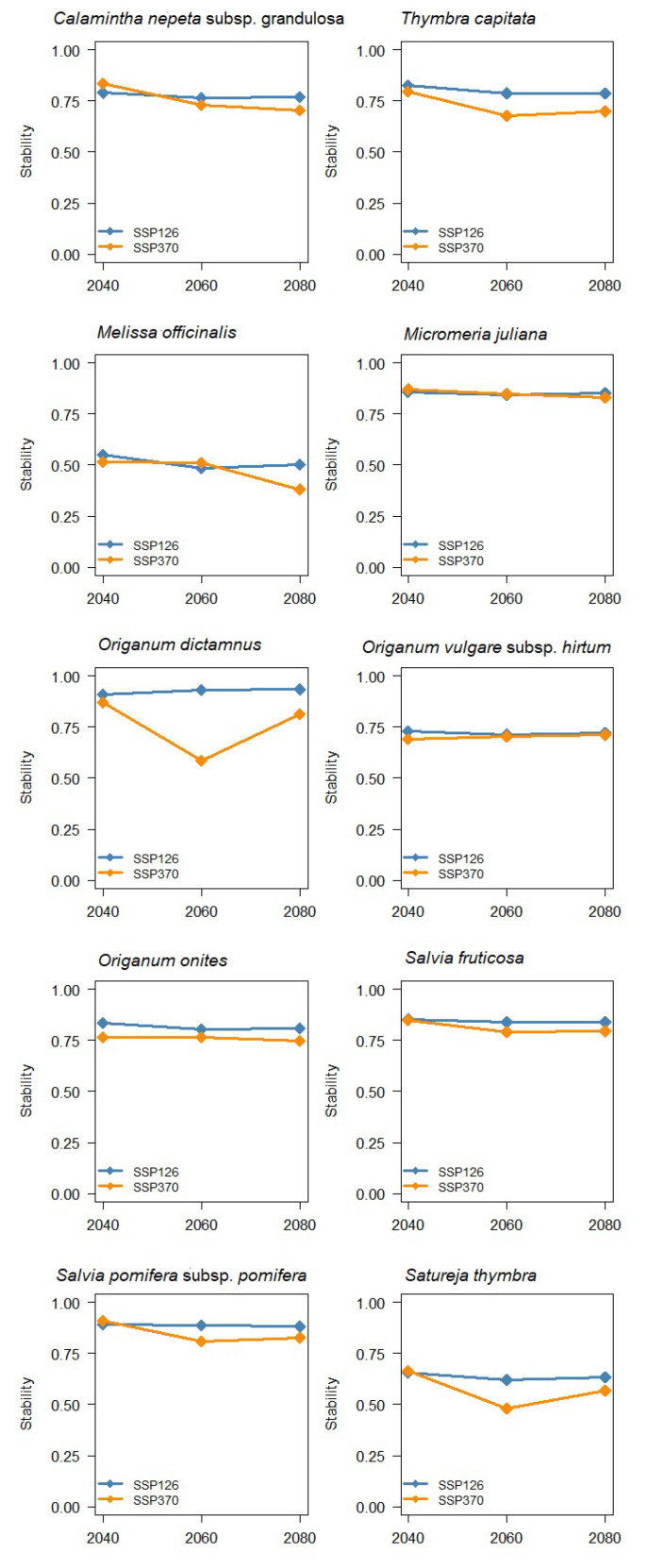
Proportion of unchanged cells (Stability Index) for every species based on the MIROC-6 climatic model and the two Shared Socio-Economic Pathways (SSP126 and SSP370).

The above classification of species into three groups also results from the stacked area plots of calculated cell change statistics for different years and climatic scenarios for both Shared Socio-Economic Pathways (Figure 5), where the portion of cells turned to suitable, remained suitable, remained unsuitable, and turned to unsuitable is quantitatively presented. In addition, the Stability Index (Figure 6) remains in relatively high values for all species except for the disadvantaged group members.

**Figure 7:**
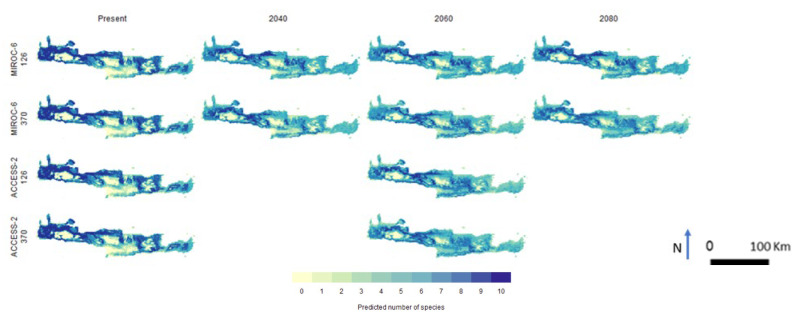
Predicted number of species for the present and future, based on different climatic models and two Shared Socio-Economic Pathways.

The predicted number of species (Figure 7) for the present varies across the island, with the lower values distributed in mountain areas. The main variation between present and future projections is estimated to be the significant reduction in areas with high numbers of species in the eastern and central parts of the island and their rarefaction in the western part, while the mountain areas remain with close to zero number of species in all future projections.

## 4. Discussion

There is an undeniable interest in studying the effects of future climate change in several aspects of plant conservation and agriculture [54,55,56]. Such studies are necessary to predict the stability of the environment in new potential initiatives, for example, the ex situ cultivation of wild medicinal plants in the frame of precision agriculture. Our results on the climate change dependencies of ex situ cultivation of ten wild medicinal taxa reveal species-dependent effects. Three groups of species were identified, the favored, the disadvantaged, and the intermediate group, concerning gains and losses in areas of potential cultivation. These data can be directly utilized for possible management implications at the regional level to help individual practitioners.

A species-dependent response of medicinal plants to climate change scenarios is supported by other studies for other areas and biomes, such as in Indonesia [57]. Gains and losses of predicted areas of occurrence for medicinal plants under future climatic conditions, which are in line with a species dependency concept, were also reported for several regions, such as Nepal [58], South Africa [59], and Egypt [60]. You et al. [61] reported that *Rhodiola* species would increase their habitat distribution in Asia, and Zhang et al. [62] suggested that under two kinds of SSPs, *Ephedra sinica* will increase its total suitable areas significantly, while Guo et al. [63] showed that under future climate scenarios, high-quality habitats of *Schisandra sphenanthera* will continue to decrease and draw near to extinction. This indicates that the species dependency response covers all the predicted distribution range shifting, from the expansion to the reduction or even extinction of the species from a given area.

On the other side, our results suggest that the species-dependent responses to climate change scenarios can be classified into groups, which likely indicates functional similarities between members within each group. As the species examined at the studied scale are phylogenetically close, being members of the Subfamily Nepetoidae Tribe Menthae [64], and well adapted to the Mediterranean environment, functional similarities, and divergences should probably be investigated at a finer scale of traits (species-level plant functional attributes), including the secondary metabolites and/or the biochemical pathways for their biosynthesis [65] and not at the scale of traditional functional divisions, e.g., tree vs. shrub, or deciduous vs. evergreen [66]. Of course, a broader set of species is required in future studies to achieve this target.

Range shifts of individual species or groups of species due to climate change have long been reported [67], and altitudinal and latitudinal variation seems to be among the dominant worldwide patterns [68]. According to this pattern, poleward and upward shifts were the main expansions of species, or species shifts predicted in response to climate change [69]. Here, the reported expansions and retractions, which are in close agreement with other types of range shifts beyond the mountain-latitude scheme, are possibly linked to complex interactions between temperature and precipitations in the landscape of the island. Mountain areas of the island remain unsuitable in all future climate scenarios and under both Socio-Economic Pathways for all the studied species, independently of their position in the classification scheme of the current study. Lowland range shifts presented here follow a longitudinal variation, where both expansions and retractions also take place.

Of course, the projected range shifts of the studied species would be used by practitioners in the decisional processes of cultivation but do not represent the only parameter of the suitability of species concerning climate change or the suitability of the farmland. Other ecological characteristics and non-climate factors may influence the species’ suitability and the suitability of the farmlands, respectively. Both of them should be additionally explored before the application of cultivation planning. For example, the effects of climate change on the quality of medicinal plants concerning the accumulation of phytochemicals (secondary metabolites) and their biosynthetic pathways remain poorly understood [70,71,72,73]. On the other side, despite the climate suitability of an area, land transformations due to past management practices may constitute lands unsuitable for cultivation. Additionally, our methodology was centered on species-level data. Factors such as genetic diversity within species, the population structure of the species on the island, or potential metapopulation structures were not considered and could be part of future studies. Thus, these factors ought to be recognized as potential limitations of the study.

Concerning plant secondary metabolic responses to global climate change, a general scheme of high management value has been suggested by Sun et al. [74]. This could be implemented in parallel to the findings of the current study by potential farmers as a guideline in the decision-making process. According to this scheme, the phenolic and terpenoid levels generally respond in a positive way to elevated carbon dioxide (eCO_2_) but negatively to elevated nitrogen deposition (eN). In addition, the total alkaloid concentration increases remarkably by eN. In contrast, decreased precipitation (dP) promotes the levels of all secondary metabolites, while elevated temperature (eT) exclusively exerts a positive influence on the levels of phenolic compounds.

## 5. Conclusions

Actions to address the consequences of climate change can be developed at two management levels: the higher level focuses on improvement actions, while the lower level centers on adaptation actions. At the higher tier, international agreements guide governments towards implementing policies that reduce greenhouse gas emissions. Meanwhile, at the lower level, adaptation actions are employed to diminish the vulnerability of specific sectors to the effects of climate change [75].

Within this framework, the cultivation of wild medicinal plants, both as a component of the agriculture sector and an ex situ conservation strategy, requires adaptation actions. The findings of our study align with this demand, emphasizing the need for enhanced cultivation planning.

## Data Availability

Not applicable.

## Supplementary Materials

The following supporting information can be downloaded at: https://www.mdpi.com/article/10.3390/biology12101327/s1, Figure S1: Predictive performance as indicated by mean AUC.
