# Supplementary material for: Climate Change Dependence in Ex Situ Conservation of Wild Medicinal Plants in Crete, Greece
Source: Biology (Basel). 2023 Oct 11;12(10):1327. doi: 10.3390/biology12101327 (PMC10604457; doi:10.3390/biology12101327)
Supplement: Supplementary file 1 [file biology-12-01327-s001.zip › biology-2617175-supplementary.pdf]

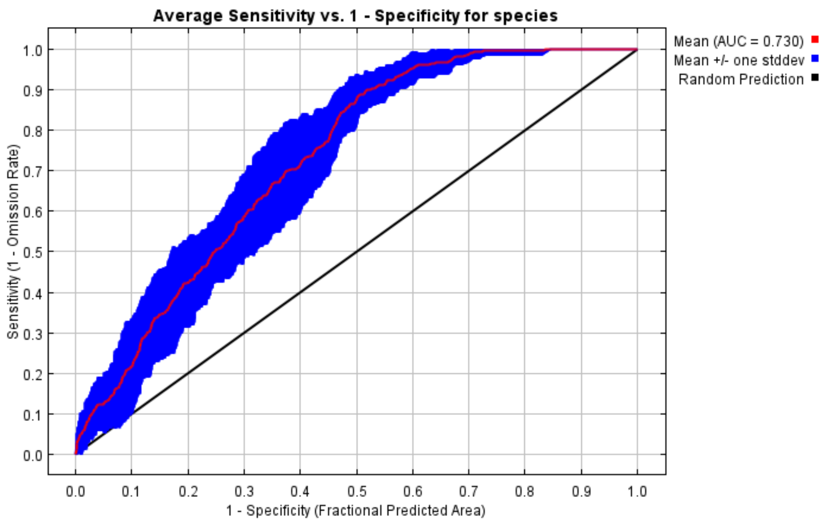

Thymbra capitata

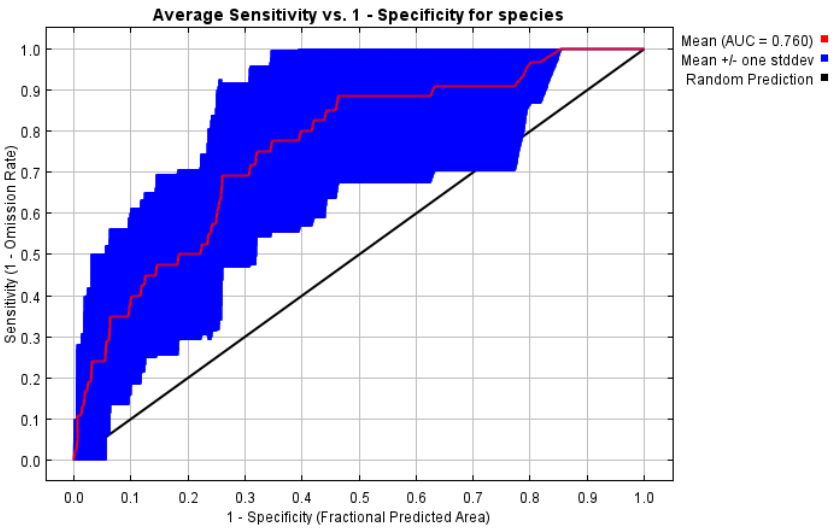

Micromeria Juliana

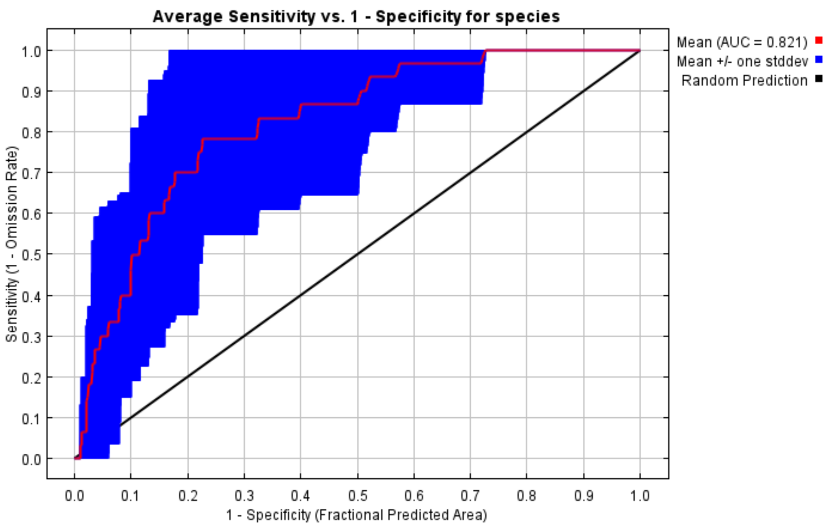

Origanum onites

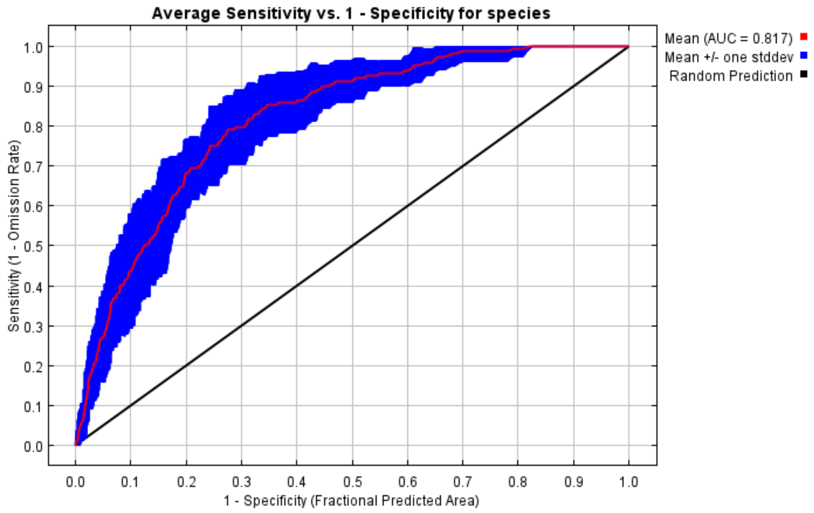

Salvia fruticosa

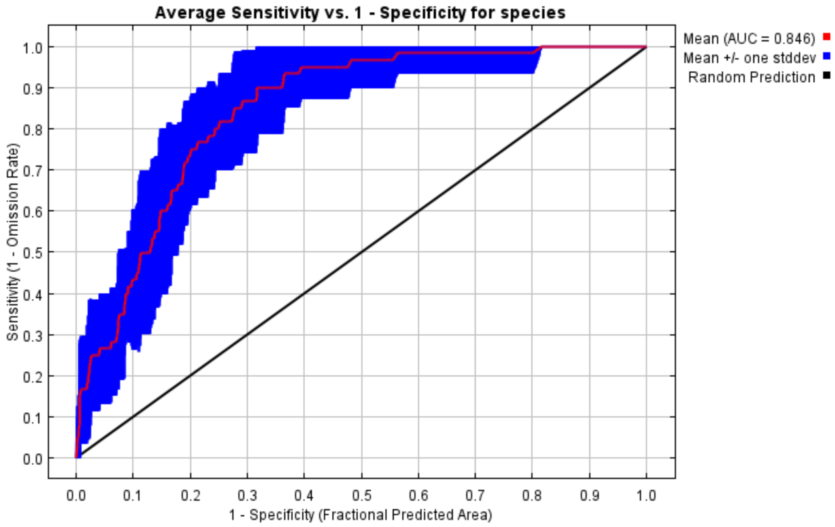

Melissa officinalis

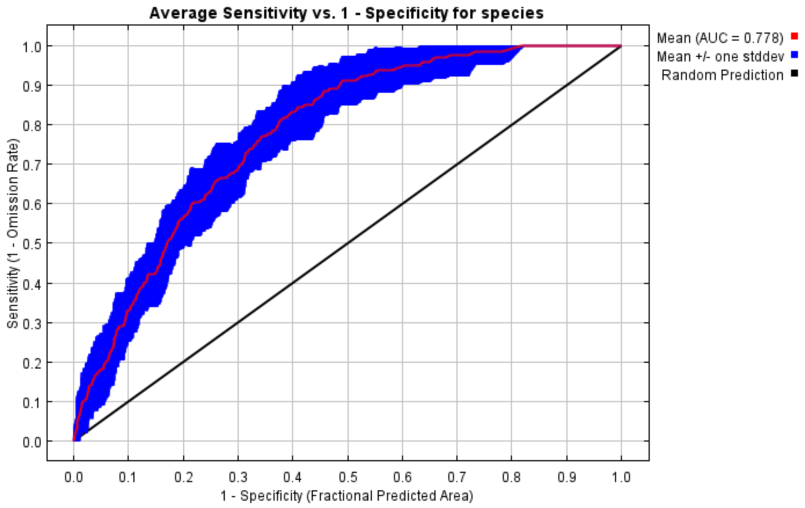

Satureja thymbra

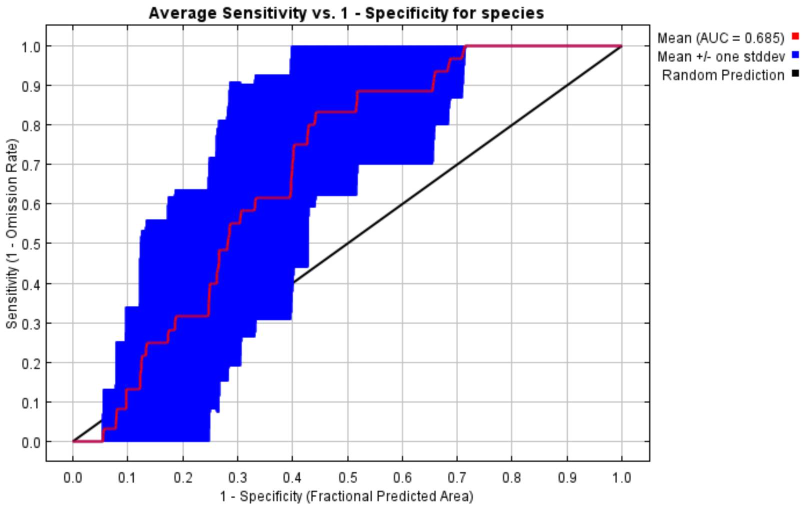

Origanum dictamnus

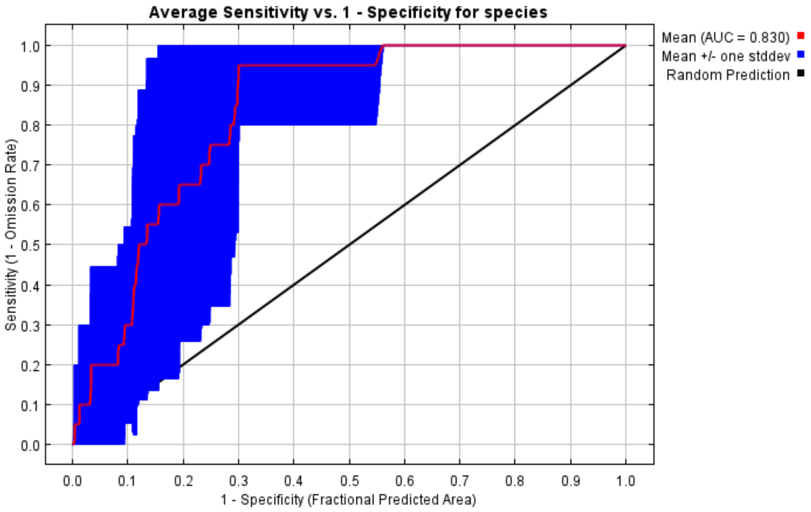

Salvia pomifera subsp. *pomifera*

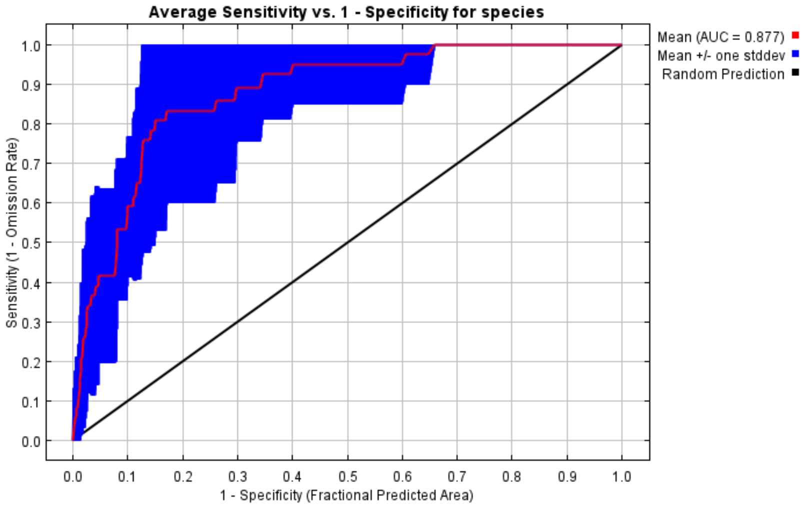

Calamintha nepeta subsp. *glandulosa*

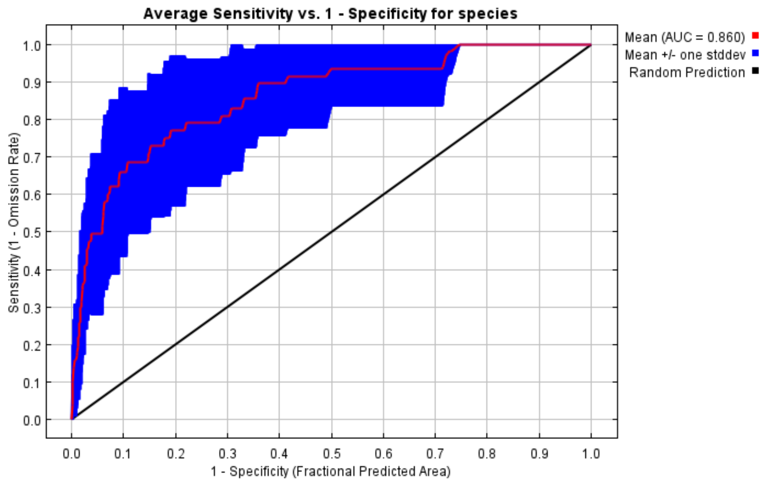

Origanum vulgare subsp. *hirtum*

Figure S1. Predictive performance as indicated by mean AUC.
